# Hyaluronic acid versus dexamethasone for the treatment of recurrent aphthous stomatitis in children: efficacy and safety analysis

**DOI:** 10.1590/1414-431X20209886

**Published:** 2020-06-26

**Authors:** Zheng Yang, Miaojuan Li, Lin Xiao, Zhiming Yi, Min Zhao, Shengjie Ma

**Affiliations:** 1 Department of Stomatology, Jiangmen Maternal and Child Health Hospital (Jiangmen Children's Hospital), Jiangmen, Guangdong, China; 2 Prevention and Healthcare, Jiangmen Municipal Stomatological Hospital, Jiangmen, Guangdong, China; 3 Department of Stomatology, Jiangmen Pengjiang Pinjie Dental Clinic, Jiangmen, Guangdong, China

**Keywords:** Dexamethasone, Hyaluronic Acid, Oral ulcer, Aphthous stomatitis, Aphthous ulcer

## Abstract

The objective of this study was to compare the safety and efficacy of 0.2% hyaluronic acid (HA) topical gel and dexamethasone topical ointment in the treatment of recurrent aphthous ulcers (RAU) in children. This retrospective observational study included 104 patients who had more than two episodes of oral aphthous ulcers per year and were treated with HA (n=52) or dexamethasone (n=52) from August 15, 2014 to September 3, 2018. Therapy efficacy was evaluated based on the ulcer size and pain score before versus 7 days after either therapy. The paired *t*-test, chi-squared test, and independent *t*-test were utilized for statistical analyses. There was no significant difference in ulcer size or pain score between the HA and dexamethasone groups, on day 1 or day 7. Both treatments were tolerated well and no side effects were reported. No significant differences in body temperature, respiration rate, pulse, or systolic/diastolic blood pressure were observed between the start (day 1) and end of treatment (day 7), for either treatment. HA and dexamethasone showed similar efficacy in reducing ulcer size and pain scores, and were tolerated equally well in children with RAU. Future high-quality studies with larger numbers of patients are needed to confirm our findings.

## Introduction

Recurrent aphthous stomatitis/ulcers (RAU) are the most common type of recurrent oral ulcers (1). RAU is an inflammatory disorder characterized by painful ovoid or round mouth ulcers that recur unpredictably (2). The condition generally presents in childhood and the prevalence in children is high, at 40% (3,4).

Aphthous ulcers (AU) can negatively impact a child's quality of life by interfering with eating and speaking, and may also result in poor school attendance (5). Vitamin deficiencies, infection, and immunodeficiency are the most common risk factors for AU in children (6,7). The pain intensity and psychological distress associated with AU vary, and the priority of therapy should be to improve the patient's quality of life (5,8).

Hyaluronic acid (HA) is a hygroscopic polymer of glucuronic acid N-acetylglucosamine disaccharide (1). It is present in mammalian body fluids and numerous tissues, such as skin and connective tissues (9). HA modulates tissue healing and can trigger the inflammatory response; it also promotes cell migration and proliferation, as well as angiogenesis (10). To our knowledge, no clinical study has assessed the safety and efficacy of topical HA in children.

Dexamethasone is a glucocorticoid utilized as a topical ointment to treat RAU and many other inflammatory disorders. It reduces inflammation by suppressing polymorphonuclear leukocyte migration and reducing capillary permeability (2,11) and has shown to be safe and effective in a randomized trial of adults (2).

The objective of this retrospective study was to compare the safety and efficacy of 0.2% HA topical gel with dexamethasone topical ointment in the treatment of RAU in children.

## Material and Methods

### Ethics approval and informed patient consent

This retrospective cohort study was conducted in the pediatric department of Jiangmen Maternal and Child Health Hospital from August 15, 2014 to September 3, 2018. Approval was received from the institutional review board of the hospital. Patient confidentiality was strictly maintained. The review followed the guidelines set forth in the Helsinki Declaration (12). Informed consent was obtained from parents or legal guardians, as applicable. The study adhered to the laws of China regarding research.

### Study population

The study population comprised patients under 18 years of age (i.e., “children” as defined by our institutional guidelines), who had experienced more than two episodes of oral AU per year and were treated on an outpatient basis with either 0.2% HA topical gel or dexamethasone topical ointment at the hospital.

### Data collection

Patients' clinical and demographic data were obtained from their medical records (chart review). Patients were diagnosed based on the findings of clinical examinations. The exclusion criteria included serious systemic disorders, requirement for systemic treatment, usage of any other ulcer medications in the previous week, and usage of systemic drugs/local medications during therapy.

### Cohort

Patients in group DX applied dexamethasone on AU three times per day (after meals) for 5 days. Patients in group HA applied topical 0.2% HA gel twice per day (after breakfast and dinner). A standard-size applicator was used.

### Outcome measurements

Therapeutic efficacy was determined based on the ulcer size and pain score at day 1 (before treatment) and 7 days after treatment. A calibrated periodontal probe was used to measure the maximum diameter and maximum width of the ulcers. The measurements were multiplied to derive the ulcer cross-sectional area. A numeric rating scale (NRS; score range: 0–10) was used to evaluate the ulcer pain. Patients were monitored for adverse reactions throughout the study (2).

### Statistical analysis

Data are reported as percentages for categorical variables, and as means and standard deviations for continuous variables. All data were analyzed using SPSS Statistics for Windows (version 21.0; IBM Corp., USA). Student's *t*-test was used for analyzing continuous variables and non-parametric tests were used for comparing the groups. P values <0.05 were considered statistically significant.

## Results

One hundred and four patients were enrolled in the present study and 52 patients were assigned to each of the two groups. The clinical and demographic data of the patients were recorded and compared between the groups, including age (range: 9–18 years), gender, ulcer type, family history of RAU, body temperature, respiration rate, pulse, and systolic/diastolic blood pressure (all P>0.05; Table 1).

**Table 1:** Clinical and demographic characteristics of children with recurrent aphthous ulcers treated with dexamethasone or hyaluronic acid.

Characteristics	Dexamethasone	Hyaluronic acid	*t* value	P value
Age (years)	13.23 (4.14)	12.54 (3.42)	0.439*	0.822
Female, n (%)	30 (57.69%)	34 (65.38%)	0.092^+^	0.420
Ulcer type, n (%)				
Minor	52 (100%)	52 (100%)	-	-
Major	0 (0)	0 (0)	-	-
Herpetiform	0 (0)	0 (0)	-	-
Family history, n (%)				
None	36 (69.23%)	33 (63.46%)	0.388^+^	0.534
1st degree	11 (21.15%)	13 (25%)	0.217^+^	0.642
2nd degree	5 (9.62%)	6 (11.54%)	0.102^+^	0.749
Temperature (°C)	36.54 (0.28)	36.62 (0.27)	1.263*	0.142
Respiration rate (breaths/min)	18.33 (1.54)	18.35 (1.63)	0.295*	0.624
Pulse (beats/min)	73.92 (6.23)	73.24 (6.47)	−0.783*	0.467
Systolic blood pressure (mmHg)	110.29 (11.03)	110.62 (11.54)	0.271*	0.324
Diastolic blood pressure (mmHg)	72.46 (7.48)	72.78 (8.11)	0.286*	0.472

Data are reported as means and standard deviations or number (n) and percent for n=52 children/group (*t-test; ^+^chi-squared test).

Patients in the DX group showed a significant reduction in ulcer size by day 7 (mean: 0.96 mm^2^) compared to day 1 (mean: 5.072 mm^2^, P<0.05; paired *t*-test). Significant pain score reduction was noted on day 7 (mean: 0.216) compared to day 1 (mean: 5.612, P<0.05; paired *t*-test) (Tables 2 and 3).

**Table 2:** Ulcer size variation of children with recurrent aphthous ulcers treated with dexamethasone or hyaluronic acid.

Days	Dexamethasone	Hyaluronic acid	P value
Day 1	5.072 (3.134)	4.616 (3.452)	0.562
Day 7	0.960 (1.698)	1.124 (1.726)	0.251
Day 1 to Day 7	4.132 (3.254)	3.508 (3.182)	0.627

Data are reported as mean and standard deviations in mm^2^ for n=52 children/group (*t*-test).

**Table 3:** Comparison of numeric rating scale variation for pain in children with recurrent aphthous ulcers treated with dexamethasone or hyaluronic acid.

Days	Dexamethasone	Hyaluronic acid	P value
Day 1	5.612 (1.584)	4.904 (1.374)	0.617
Day 7	0.216 (1.027)	0.668 (1.143)	0.192
Day 1 to Day 7	5.183 (1.656)	4.223 (1.494)	0.388

Data are reported as means and standard deviations for n=52 children/group (*t*-test). Numeric rating scale (score range: 0-10).

In the HA group, ulcer size showed a significant reduction by day 7 (mean: 1.124 mm^2^) compared to day 1 (mean: 4.616 mm^2^, P<0.05; paired *t*-test). A significant reduction in the pain score was also noted on day 7 (mean: 0.668) compared to day 1 (mean: 4.904, P<0.05; paired *t*-test).

There was no significant group difference in ulcer size or pain score, on day 1 or day 7.

Both treatments were well tolerated by all patients and none reported any side effects. No significant group differences in body temperature, respiration rate, pulse, or systolic/diastolic blood pressure were observed between the start (day 1) and end (day 7) of the treatment period (Table 4).

**Table 4:** Comparison of characteristics of the two groups of children with recurrent aphthous ulcers treated with dexamethasone or hyaluronic acid after 7 days of treatment.

Characteristics	Dexamethasone	Hyaluronic acid	*t* value	P value
Temperature (°C)	36.51 (0.32)	36.59 (0.24)	1.139	0.342
Respiration rate (breaths/min)	18.28 (1.49)	18.38 (1.58)	0.565	0.463
Pulse (beats/min)	73.86 (5.94)	73.07 (6.12)	−0.678	0.594
Systolic blood pressure (mmHg)	109.70 (10.42)	109.84 (9.48)	0.218	0.802
Diastolic blood pressure (mmHg)	71.98 (6.64)	72.12 (7.85)	0.614	0.536

Data are reported as means and standard deviations for n=52 children/group (*t*-test).

## Discussion

Nearly 40% of patients with RAU have a family history of the condition. It usually occurs in early childhood, with episodes gradually increasing in frequency (4,13). In the present study, 33.65% of patients had a family history of RAU. Morphologically, RAU is categorized as a minor, major, or herpetiform ulcer type. The minor form of RAU accounts for 80% of cases, and is characterized by lesions measuring 5–10 mm on labial and buccal surfaces, as well as the labial mucosa, which resolve spontaneously within 7–10 days with no scarring (2–4). All the patients in the current study had the minor form of RAU.

There is no established management guideline for RAU, and there are numerous treatment options. No study has assessed the efficacy of the various treatment methods in children (5).

Dexamethasone is a glucocorticoid utilized as a topical treatment for RAU in China; it is also used to treat inflammatory lesions such as chronic ulcers, gingival ulcers, and erosive stomatitis. Dexamethasone alleviates inflammation by suppressing polymorphonuclear leukocyte migration and reducing capillary permeability (2,11). A randomized placebo-controlled study conducted by Liu et al. (2) including 240 adult patients indicated that dexamethasone was safe and effective for treating RAU; significant decreases in ulcer size and pain scores on day 6±2 post-therapy were observed, without adverse effects. The present study outcomes are in agreement with the above adult study.

HA plays a key role in the growth, development, and repair of tissues (14). A randomized placebo-controlled study by Nolan et al. (15) demonstrated the efficacy of a topical HA (0.2%) preparation in managing RAU in 120 adult patients; significantly fewer ulcers were observed on day 5 compared to the placebo group. Additionally, significantly fewer ulcers recurred in patients treated with HA on day 4 compared to patients in the placebo group. A prospective study by Lee et al. (16) also showed the efficacy and safety of topical 0.2% HA gel in the treatment of 33 adult patients with Behçet's disease or RAU.

In the present study, dexamethasone ointment and 0.2% HA topical gel showed similar efficacy and safety, similar to the results of prior adult studies (2,15,16). The current retrospective study, to our knowledge, is the first to examine the efficacy of these treatment modalities in children with RAU.

Although the risks associated with corticosteroid use can be mitigated by limiting the treatment duration, corticosteroids are contraindicated in certain patients. HA provides faster pain relief than corticosteroid ointment, irrespective of ulceration stage, and is associated with a lower risk of complications, discomfort, and drug interactions. There is no overdose risk with HA, and it is often safer than corticosteroids for younger children who may have difficulty following directions. Additionally, it is widely available over the counter (2,15,16).

Although the outcomes of this study are encouraging, there were some limitations. First, the study used a retrospective, single-center design and included a very limited patient population; moreover, there was no placebo group. Furthermore, lack of follow-up may have introduced selection and information biases. Future studies with a larger number of patients should address the above limitations and compare RAU treatment protocols based on antibiotics, corticosteroids, and other anti-inflammatory drugs in addition to HA.
